# Second opinion needed: communicating uncertainty in medical machine learning

**DOI:** 10.1038/s41746-020-00367-3

**Published:** 2021-01-05

**Authors:** Benjamin Kompa, Jasper Snoek, Andrew L. Beam

**Affiliations:** 1 Department of Biomedical Informatics, Harvard Medical School, Boston, MA USA; 2 Google Brain, Cambridge, MA USA; 3 Department of Epidemiology, Harvard T.H. Chan School of Public Health, Boston, MA USA

**Keywords:** Health care, Medical research

## Abstract

There is great excitement that medical artificial intelligence (AI) based on machine learning (ML) can be used to improve decision making at the patient level in a variety of healthcare settings. However, the quantification and communication of uncertainty for individual predictions is often neglected even though uncertainty estimates could lead to more principled decision-making and enable machine learning models to automatically or semi-automatically abstain on samples for which there is high uncertainty. In this article, we provide an overview of different approaches to uncertainty quantification and abstention for machine learning and highlight how these techniques could improve the safety and reliability of current ML systems being used in healthcare settings. Effective quantification and communication of uncertainty could help to engender trust with healthcare workers, while providing safeguards against known failure modes of current machine learning approaches. As machine learning becomes further integrated into healthcare environments, the ability to say “I’m not sure” or “I don’t know” when uncertain is a necessary capability to enable safe clinical deployment.

## Introduction

There has been enormous progress towards the goal of medical artificial intelligence (AI) through the use of machine learning, resulting in a new set of capabilities on a wide variety of medical applications^1–3^. As these advancements translate into real-world clinical decision tools, many are taking stock of what capabilities these systems presently lack^4^, especially in light of some mixed results from prospective validation efforts^3,5,6^. While there are many possibilities, this article advocates that uncertainty quantification should be near the top of this list. This capability is both easily stated and easily understood: medical ML should have the ability to say “**I don’t know**” and potentially **abstain** from providing a diagnosis or prediction when there is a large amount of *uncertainty* for a given patient. With this ability, additional human expertise can be sought or additional data can be collected to reduce the uncertainty to make a more informed diagnosis.

Indeed, communicating uncertainty and seeking a second opinion from colleagues when confronted with an unusual clinical case is a natural reflex for human physicians. However, quantification and communication of uncertainty is not routinely considered in the current literature, but is critically important in healthcare applications. For instance, four of the most widely cited medical ML models published since 2016 do not have a mechanism for abstention when uncertain^7–10^ and do not report sample level metrics such as calibration, echoing what has been observed in systematic meta-analyses^11^. This more cautious approach to medical ML will allow safer clinical deployment and help engender trust with the human healthcare workers who use this technology, since they will have the ability to know when the model is and is not confident in the diagnostic information it is providing.

In healthcare applications, machine learning models are trained using patient data to provide an estimate of a patient’s *current* clinical state (diagnosis) or *future* clinical state (prediction). Though diagnostic and prognostic classification models estimate the same statistical quantity (i.e., the conditional probability of a clinical state or event), diagnosis and prognosis differ greatly in their interpretation and use cases^12^. To complicate matters further, it is common in the machine learning literature to refer to any *point estimate* (i.e., the model or algorithm’s “best guess”) of this type as a “prediction”^13^. There are also at least two types of uncertainty quantification worth considering. The first, and most straightforward, is to consider the point-estimate of the conditional probability provided by the model as an indication of the model’s confidence: extremely low or extremely high probabilities indicate high confidence while probabilities near 0.5 indicate a lack of confidence. If these models are also calibrated, then the predicted probability of an outcome corresponds to the observed empirical frequency. Model calibration is well studied in the traditional medical stats and epidemiology literature^14–18^. A second kind of uncertainty acknowledges that the point-estimate itself could be unreliable and seeks to estimate the *dispersion* or *stability* of this point estimate. Estimating this is kind of uncertainty for complicated machine learning models can be quite challenging and is an active area of research. For the purposes of this discussion, we will use the term *predictive uncertainty* to refer to the stability of a point estimate provided by the model to better align with the larger machine learning literature. We will also discuss how the point estimate itself (i.e., the conditional probability) can be used as a reasonable measure of uncertainty in certain scenarios. Finally, not all healthcare events are binary or categorical, but we will mostly restrict the discussion to classification tasks while acknowledging that these ideas apply equally well to regression scenarios.

## What is uncertainty quantification?

The quantification and communication of uncertainty from predictive models is relatively common in everyday life. For instance, weather forecasts provide excellent examples of uncertainty estimates. Hurricane forecasts provide not only the most likely point of landfall, but also provide a “cone of uncertainty” across other likely points of impact and future trajectories of the storm. Using this information, officials can make more informed preparations and prepare safer evacuation plans.

In contrast, most of the ML systems in the recent medical literature neglect predictive uncertainty. This is analogous to a hurricane forecast only providing the single, most likely point of landfall, which would make storm preparations extremely difficult. This example illustrates the crucial point: a model that provides predictive uncertainty information allows for better decision making and planning.

**Fig. 1:**
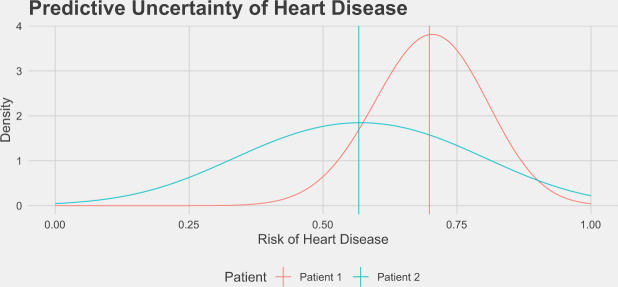
Predictive uncertainty for the risk of heart disease in two patients. These distributions of risks over models were generated by randomly bootstrapping 1000 datasets from the Heart Disease UCI dataset^19^ and training logistic regression models on each dataset. These distributions are the range of risks from this class of model assigned to these patients when they occurred in the test set, and the mean risk from the full dataset are shown as vertical lines. Despite the fact that both patients have similar mean risks for heart disease, we may be more inclined to trust the predictions for patient 1 given the lower amount of uncertainty associated with that prediction.

To illustrate predictive uncertainty in a classification setting, we bootstrapped the predicted risk of heart disease for two patients on the basis of clinical features such as age, sex, smoking status, cholesterol, blood pressure, etc^19^, and the distribution of these scores is displayed in Fig. 1. The mean risk estimated using the full dataset for each patient is indicated by the vertical line at 55 and 65%, respectively. It is clear graphically that the predictive uncertainty for these two patients is quite different, as the distribution of likely scores for patient 1 is much more dispersed than the distribution for patient 2. One way to quantify the predictive uncertainty would be to calculate the standard deviation of these empirical distributions, which are 7.6% and 15.3% for patient 1 and patient 2, respectively. Using this information, we could flag patient 2 as needing more information before making a clinical decision.

## What are the sources of uncertainty?

Predictive uncertainty stems from multiple sources of missing information, bias, and noise^20,21^. First, there can be noise in data measurement and this has recently become known as *aleatoric* uncertainty in the machine learning literature. This type of uncertainty is *irreducible* and can not be resolved by collecting more data. Additionally, there is uncertainty in the estimated model parameters and indeed over which model to even select in the first place. These last two factors contribute to *epistemic* uncertainty^20,21^

There is also the strong possibility of *dataset shift* when deploying a model in practice. Dataset shift can take many forms^22,23^. In general, it consists of changes in the distributions of either *Y*, the data labels, or *X*, the data features, between the training and testing datasets. For instance, covariate shift is when the distributions of the training dataset features and testing dataset features differ but the conditional distribution of the data labels given the input data is equivalent for both datasets^22^. Label shift is the opposite effect, when data label distributions differ but the conditional distributions of the input features given the label are the same^22^. There are additional dataset shift effects that can be quite subtle but important to consider in practice. Dataset shift is an important component of predictive uncertainty in practice. Ovadia et al.^24^ performed an extensive benchmark of the effects of dataset shift on deep learning methods’ uncertainty estimates and this study is described in more detail below.

## What are some ways to calculate predictive uncertainty?

Calculating predictive uncertainty for a new observation depends heavily on the underlying model. Despite the variety of models available, many different uncertainty quantification techniques capture the same notion: the distance of the new observation to observations it has previously seen. In order to learn the parameters of a model, researchers leverage a training dataset. Then, a test dataset is used to evaluate performance on unseen data. Just as a patient with a unique presentation will cause uncertainty in a physician’s diagnosis, a test point far from training data should result in a higher amount of predictive uncertainty. Over the next section, we survey several methods to calculate predictive uncertainty. These include prediction intervals, conformal sets, Monte Carlo dropout, ensembling, and several Bayesian methods including Gaussian processes.

One classic way to provide predictive uncertainty for linear regression is through a 95% prediction interval, which can be calculated by^25^:

1 $$\begin{array}{*{20}{c}} {\mathop{y}\limits^{\frown} \pm t_{n - 2}^ \ast s_y\sqrt {1 + \frac{1}{n} + \frac{{(x_{n + 1} - \bar x)^2}}{{\left( {n - 1} \right)s_x}}} } \end{array}$$

where $\mathop{y}\limits^{\frown}$ is the predicted *y* from the linear regression model, t^*^ is the critical value from the *t-*distribution, *n* is the size of the training set, *s*_*y*_ is the standard deviation of the residuals, and $\bar x$ is the sample mean and *s*_*x*_ is the sample standard deviation, respectively. The distance from the training data is captured by the $(x_{n + 1} - \bar x)^2$ term. This is the squared distance of our new sample *x*_*n* + 1_ from the mean of the training data. With this formulation, the true *y* for *x*_*n* + 1_ will fall in this range 95% of the time, on average, after many repeated studies. Unfortunately, the assumptions needed for these coverage guarantees are violated by more complicated machine learning models and are not easily extended to classification models.

However, with an approach known as conformal inference^26^, it’s possible to obtain exact *marginal* coverage guarantees per sample for virtually any standard machine learning model in both regression and classification settings. This is improved over the guarantees from the above prediction intervals since rather than averaging over many collections of data, marginal guarantees are satisfied in finite samples. More precisely, if we let *C*(*x*_*n* + 1_) be the conformal set of predictions for a sample *x*_*n* + 1_, then having a marginal coverage guarantee would mean:

2 $$\begin{array}{*{20}{c}} {P\left( {y_{n + 1} \in C\left( {x_{n + 1}} \right)} \right) = 1 - \alpha } \end{array}$$

So the true label *y*_*n* + 1_ is in the predicted set with probability 1−*α* averaged over the entire dataset. Note that conformal inference allows us to leverage (potentially uncalibrated) point estimates from a machine learning classifier and produce conformal sets with the desired coverage properties. Predictive uncertainty in this case would be the size of the conformal set: if the set contains both the healthy and disease class we may trust the prediction on this particular sample less.

Ideally, there could be distribution free *conditional* guarantees which would be true for any given sample *x*_*n* + 1_; however, this is not possible in general^27^. Conditional guarantees would mean:

3 $$\begin{array}{*{20}{c}} {P\left( {y_{n + 1} \in C\left( {x_{n + 1}} \right){\mathrm{|}}x_{n + 1} = x} \right) = 1 - \alpha } \end{array}$$

Then the true label is in the predicted set with probability 1−*α* for *this specific* data point. The difference between marginal and conditional coverage is like giving a patient an average 5-year survival rate for those affected with their cancer versus given a predicted 5-year survival rate for that specific patient based on their personal clinical features. Unfortunately, general conditional guarantees are not possible in conformal inference^27^.

Conformal inference relies on the notion of distance from the training data through a “nonconformity score”. An example nonconformity score for classification tasks could be 1 minus the predicted probability of the positive class. New test points and their accompanying model predictions have a nonconformity score calculated and compared to the empirical distribution of the nonconformity scores of a held-out portion of the training data. In this way, model predictions are accepted or rejected into the conformal prediction set or interval. Conformal inference also is not generally robust to dataset shift. However, recent work by Barber et al. extends conformal inference guarantees to the setting of covariate shift^28^.

For neural networks and deep learning methods, some simple methods to calculate conditional uncertainty estimates include Monte Carlo (MC) Dropout^29^ and ensembling^30–32^. MC Dropout consists of randomly removing hidden unit outputs at train and/or test time in a neural network. Outputs in the neural network architecture are set to 0 with probability *p* according to a Bernoulli distribution^29^. A prediction is made by randomly sampling different configurations and then averaging across these different dropout realizations. MC Dropout was initially introduced as an ad hoc modification to neural networks^20^, but since then have been shown to be an approximation of Bayesian variational inference under a certain set of assumptions^29^. Ensembling is a flexible method that can be applied to a variety of machine learning models^33^. For neural networks, ensemble methods require training multiple networks on the same data then combining predictions from these networks, resembling bootstrap procedures from the statistical literature. In ensembles of *M* deep neural networks, predictions from the different models are averaged^30^. Predictive uncertainty from both MC Dropout and ensembling can be summarized by calculating the standard deviation (or similar metric of dispersion) from the collection of predictions provided by each approach. Both methods are easy to add to existing neural network models and provide good uncertainty estimates on out of distribution data^24^.

Bayesian methods to calculate predictive uncertainty estimates generally rely on the posterior predictive distribution:

4 $$\begin{array}{*{20}{c}} {p\left( {y\left| {X,D} \right.} \right) = {\int} {p\left( {y{\mathrm{|}}X,W} \right)p\left( {W{\mathrm{|}}D} \right)dW} } \end{array}$$

where *y* is the outcome of interest (i.e. heart disease status), *X* is the data for a specific sample (i.e. a patient’s clinical markers), *D* is the training data of the model, and *W* are the parameters of the ML model. Once the posterior predictive distribution has been estimated, predictive uncertainty is straight-forward to obtain since one has access to the entire distribution of interest. For neural networks and many machine learning models however, calculating the posterior predictive distribution exactly is analytically intractable in general and requires computational approximations. For instance, the integral over the model weights can be replaced by an average over many samples of model weights obtained from a Markov-Chain Monte Carlo simulation^34^.

In Bayesian neural networks, much work has gone into improving approximations of *p(W* | *D)*. Being able to estimate this posterior well should allow for good uncertainty estimates based on theoretical and empirical evidence^24,35^. Variational inference methods^36,37^ are one popular class of approximations, but impose stricter assumptions about correlations between model parameters than more flexible methods^4,38–42^. However, variational inference is known to underestimate the posterior probability distribution^43^. This could have major implications for uncertainty estimates based on these approximations of the posterior. Yao et al. provides a systematic comparison across ten popular approximations^44^. Recent work by Wenzel et al.^45^ demonstrates that fundamental unresolved challenges remain to estimating p(W | D) in a manner that improves predictive uncertainty in variational inference and Bayesian neural networks more generally.

Ovadia et al. also showed in a benchmark of deep learning models under dataset shift that variational methods were difficult to use in practice and only had good uncertainty estimates on the simple datasets^24^. They assessed many models including post-hoc calibration of predictions, ensembles, Dropout, and variational methods on multiple classification datasets. Models were compared based on proper scoring rules^24,46^. Proper scoring rules are one key way to compare uncertainty estimates across different methods.

Gaussian processes are an alternative Bayesian method that have natural predictive uncertainty estimates built in. A Gaussian process defines a prior distribution over the types of functions that could fit the training data^47^. After conditioning on the actual observed training data *X*, Gaussian processes allow us to compute a normal distribution at each point of the test set X_*_:

5 $$\begin{array}{*{20}{c}} {\left[ {\begin{array}{*{20}{c}} {\mathrm{f}} \\ {f_ \ast } \end{array}} \right] \sim N\left( {0,\left[ {\begin{array}{*{20}{c}} {K\left( {X,X} \right)} & {K\left( {X,X_ \ast } \right)} \\ {K\left( {X_ \ast ,X} \right)} & {K\left( {X_ \ast ,X_ \ast } \right)} \end{array}} \right]} \right)} \end{array}$$

*f* and f_*_ are the joint normal distributions of the training and test data, respectively^47^. The means of these normal distributions are the point estimates for our test set. The variance of the normal distributions provide a natural estimate of predictive uncertainty. In the limit of infinite width, neural networks are equivalent to Gaussian processes^48–50^.

*K* is the covariance function, also known as the “kernel” function, and computes the similarity between all points in the respective sets being evaluated. One could choose the covariance function to be the Euclidean distance function and the kernel directly calculates the distance between training and test points. Common choices of kernels include periodic functions and squared exponential functions^47^. Ultimately, Gaussian processes scale poorly in the number of data points^47^ and have been challenging to apply to structured problems where a good covariance function is unknown a priori (i.e. in the case of dataset shift)^24,51^.

## How do we go from uncertainty estimation to abstention?

Uncertainty estimates naturally allow a physician to subjectively abstain from utilizing the model’s predictions heuristically. If there is high predictive uncertainty for a sample, the physician can discount or even disregard the prediction. However, there are methods that allow models to choose to abstain themselves. For instance, conformal inference methods can return the empty set for a classification task, which indicates that no label is sufficiently probable.

More generally, allowing models to abstain from prediction is known as “selective prediction.”^52^ Selective prediction models generally rely on two ideas: optimizing a model with respect to a loss function where abstention is given a specific cost or learning to abstain such that a model achieves certain performance criteria (e.g. X% accuracy with probability *δ* for some proportion of the data)^52^. These “cost-based” and “bounded” objectives are reflections of each other; abstention rules from each objective can be transformed into corresponding rules in the other objective^53^.

For instance, if one wanted to optimize a model with a 0-1 loss function with an abstain option, one could write^54^:

6 $$\begin{array}{*{20}{c}} {L\left( {Y,\hat Y} \right) = \left\{ {\begin{array}{*{20}{c}} {0\;if\;\hat Y = Y} \\ {\alpha \;if\;\hat Y = \bot } \\ {1\;if\;\hat Y \ne Y} \end{array}} \right.} \end{array}$$

where *Y* is the ground truth label for a sample,$\hat Y$ is the predicted label, and 0 ≤ *α* ≤ 1. The ⊥ symbol indicates the model abstained from prediction and decided to incur cost *α* rather than risk predicting incorrectly and incurring cost 1. Optimizing with respect to cost sensitive lost functions has been explored in many settings including binary predictions^55–58^, multiclass prediction^54^, class imbalance^53^, and deferring to experts^59^.

Bounded objectives often rely on learning a rejection function that modulates whether a model will predict or abstain for a sample. This can be formalized as:

7 $$\begin{array}{*{20}{c}} {\left( {{\mathrm{f}},{\mathrm{g}}} \right)\left( {\mathrm{x}} \right) = \left\{ {\begin{array}{*{20}{c}} {f\left( {\mathrm{x}} \right)if\,g\left( {\mathrm{x}} \right) \ge h} \\ { \bot if\,g\left( {\mathrm{x}} \right) < h} \end{array}} \right.} \end{array}$$

where *f* is a typical model and *g* is a selection function that permits *f* to predict if *g(x)* exceeds a threshold *h* and abstain otherwise.

Determining a suitable selection function is the crux of these bounded methods. Methods such as softmax response^60^ and SelectiveNet^52^ learn a selection function based on uncertainty estimates. These models rely on underlying estimates of uncertainty per sample. For highly uncertain samples, the models abstain from making a prediction. Uncertainty estimates allow these models to have low levels of risk (i.e. mean loss, see Geifman et al. 2017^60^) with high probability across large proportions of the dataset. When training a model, one can specify desired levels of risk and with what probability that risk is expected to be met. Deep Gamblers^61^ is an alternative method that leverages financial portfolio theory to learn a selection function based on uncertainty estimates and has shown improved performance relative to softmax response and SelectiveNet.

## Why do we need uncertainty estimation and abstention?

For models that predict critical conditions (e.g. sepsis), uncertainty estimates will be vital for triaging patients. Physicians could focus on patients with highly certain model estimates of critical conditions, but also further examine patients for whom the model is uncertain with respect to their current condition. For patients with highly uncertain predictions, additional lab values could be requested to provide more information to the model. Additionally, uncertainty estimates could be used to detect outliers. Patient’s data which is not represented in the training set should cause models to report high predictive uncertainty. For example, an imaging model that detects the location of organs in an MRI would have highly uncertain predictions for a patient with situs inversus (mirrored organs). Over time, well calibrated uncertainty models should earn the trust of physicians by allowing them to know when to accept the model’s predictions. Furthermore, abstention allows models to ask the downstream medical expert to take a second look at the patient. The point of abstention is not to obscure the model’s output, which could still be displayed to the end user. Instead, it is a mechanism to communicate an elevated level of uncertainty automatically and say “I don’t know” to emphasize the need for a human to look at the issue. This is one more way the uncertainty-equipped models can engender user-trust.

Uncertainty estimates could also serve as a safety measure. It’s important to understand if any dataset shift has occurred when a model is deployed to the real world. Dataset shift could occur when a model that was trained on data from one hospital is validated in a different hospital^62^. The validation hospital might have different typical ranges for many features included in the model. A properly calibrated model should report high uncertainty for input values that are outside of the typical ranges from training data.

More insidiously, there are scenarios in which an adversarial attack may be launched to modify the predictions of a medical machine learning model^63^. With very small perturbations to model input, adversarial attacks can arbitrarily change the model output. Models should provide high estimates of uncertainty in their highly confident predictions when faced with an adversarial attack.

## Conclusions

Medical ML models will be increasingly integrated into clinical practice, and incorporation of predictive uncertainty estimates should become a required part of this integration. With the ability to say “I don’t know” based on predictive uncertainty estimates, models will be able to flag physicians for a second opinion. Though it remains an open and challenging area of research, strides are being made in understanding the best ways to quantify and communicate predictive uncertainty^24,64^. These uncertainty-equipped models will be able to improve patient care, engender physician trust, and guard against dataset shift or adversarial attacks. Incorporating uncertainty estimates into medical ML models represents an addressable next-step for these models.
